# A diet based on multiple functional concepts improves cardiometabolic risk parameters in healthy subjects

**DOI:** 10.1186/1743-7075-9-29

**Published:** 2012-04-02

**Authors:** Juscelino Tovar, Anne Nilsson, Maria Johansson, Rickard Ekesbo, Ann-Margreth Åberg, Ulla Johansson, Inger Björck

**Affiliations:** 1 Antidiabetic Food Centre, Lund University, P.O. Box 121, SE-221 00 Lund, Sweden; 2 Department of Applied Nutrition and Food Chemistry, Lund University, SE-221 00 Lund, Sweden; 3 Primary Health Care Centre, Dalby, Sweden; 4 Idun Mat och Näringskonsult, Lund, Sweden

**Keywords:** Cardiometabolic disease, metabolic syndrome, dietary prevention, functional foods, randomized controlled trial, crossover design

## Abstract

**Background:**

Different foods can modulate cardiometabolic risk factors in persons already affected by metabolic alterations. The objective of this study was to assess, in healthy overweight individuals, the impact of a diet combining multiple functional concepts on risk markers associated with cardiometabolic diseases (CMD).

**Methods:**

Fourty-four healthy women and men (50-73 y.o, BMI 25-33, fasting glycemia ≤ 6.1 mmol/L) participated in a randomized crossover intervention comparing a multifunctional (active) diet (AD) with a control diet (CD) devoid of the "active" components. Each diet was consumed during 4 wk with a 4 wk washout period. AD included the following functional concepts: low glycemic impact meals, antioxidant-rich foods, oily fish as source of long-chain omega-3 fatty acids, viscous dietary fibers, soybean and whole barley kernel products, almonds, stanols and a probiotic strain (*Lactobacillus plantarum *Heal19/DSM15313).

**Results:**

Although the aim was to improve metabolic markers without promoting body weight loss, minor weight reductions were observed with both diets (0.9-1.8 ± 0.2%; *P *< 0.05). CD did not modify the metabolic variables measured. AD promoted significant changes in total serum cholesterol (-26 ± 1% vs baseline; *P *< 0.0001), LDL-cholesterol (-34 ± 1%; *P *< 0.0001), triglycerides (-19 ± 3%; *P *= 0.0056), LDL/HDL (-27 ± 2%; *P *< 0.0001), apoB/apoA1 (-10 ± 2%; *P *< 0.0001), HbA_1c _(-2 ± 0.4%; *P *= 0.0013), hs-CRP (-29 ± 9%; *P *= 0.0497) and systolic blood pressure (-8 ± 1%¸ *P *= 0.0123). The differences remained significant after adjustment for weight change. After AD, the Framingham cardiovascular risk estimate was 30 ± 4% (*P *< 0.0001) lower and the Reynolds cardiovascular risk score, which considers CRP values, decreased by 35 ± 3% (*P *< 0.0001).

**Conclusion:**

The improved biomarker levels recorded in healthy individuals following the multifunctional regime suggest preventive potential of this dietary approach against CMD.

## Background

Cardiometabolic diseases (CMD), i.e. cardiovascular disease and diabetes mellitus, are important causes of morbidity and mortality worldwide [1]. Therefore, any person with multiple cardiometabolic risk factors would benefit from lifestyle changes, including improvement of dietary habits, to favorably modify as many as possible risk-associated features [2]. Current recommendations for dietary management of subjects with high metabolic risk involve quantitative and qualitative changes in fat and sugar intakes and high consumption of fruits, vegetables, and whole grains [3]. These guidelines focus on lowering LDL cholesterol, fasting glycemia, body weight and blood pressure as a way to reduce the risk of heart disease [2,3]. In this context, a portfolio of vegetarian foods has been shown capable of improving LDL cholesterol, HDL cholesterol and CRP values in hypercholesterolemic subjects [4-7]. Similarly, a Nordic-type diet can modulate the blood lipid profile of hypercholesterolemics [8].

However, a more comprehensive approach to the dietary prevention of CMD also requires attention to other risk factors besides altered blood lipids or glycemia. An increasing number of studies show that different functional foods and ingredients [9,10] may exert positive effects on particular parameters related to the metabolic syndrome (MetS), a cluster of pathophysiologic conditions increasing cardiometabolic risk [11-13]. Such observations may provide the basis for an effective preventive dietary strategy, in which the inclusion of an ample spectrum of active food components or properties may decrease multiple risk factors. The present investigation explores the CMD preventive potential of a diet based on a combination of functional concepts, chosen on the basis of their beneficial effects on single metabolic risk markers.

Since subclinical chronic inflammation is considered an important factor in the etiology of CMD and MetS [2,12,13], the components of the multifunctional "active" diet (AD) were selected mainly for their perceived abilities to reduce inflammatory tonus. Within this frame, AD included low glycemic impact foods/meals, foods naturally rich in antioxidant polyphenols and fish products rich in omega-3 fatty acids [14-17]. Moreover, the active diet also contained a probiotic *Lactobacillus *strain capable to decrease the inflammatory response in an animal model [18] and an important supply of soluble dietary fiber from barley and oat, with suggested prebiotic antiinflammatory action in humans [19-21]. Another functional concept encompassed in the diet corresponds to foods and ingredients with recognized blood lipid-modulating actions, cholesterol lowering items in particular [2,4].

We hypothesized that a combination of the above food concepts may target sub-clinical inflammation in a synergistic way and thus allow for the prescription of practically feasible intakes of the active foods. The study was performed in a healthy middle-aged group of overweight volunteers, comparing the AD vs. a control diet (CD) using a cross-over design, with 4-wk study periods. The aim was to improve cardiometabolic risk-associated parameters by changing dietary composition without promoting weight loss.

## Participants and methods

### Participants

Volunteers without any known medical condition (36 females, 8 men) were recruited through advertisement in local newspapers and were informed orally and in writing of the project disposal, emphasizing the non weight-loss character of the investigation. Inclusion criteria were age between 50 and 73, body mass index in the 25-33 kg/m^2 ^range and fasting plasma glucose value ≤ 6.1 mmol/L. Only medications accepted were hormone replacement for thyroid problems (if constant during the whole trial; 1 female subject) and prescription-free painkillers without antinflammatory action. Baseline data collected at the time of the first clinical visit are shown in Table 1.

**Table 1:** Characteristics of participants at baseline^a^

	Whole group (n = 44)	Females **(n = 36)**^**b**^	Males (n = 8)
Age (y)	63.3 ± 0.8	63.2 ± 0.8	63.6 ± 1.9

Body weight (kg)	79.4 ± 1.4	77.4 ± 1.9	88.6 ± 2.5

BMI (kg/m^2^)	28.5 ± 0.3	28.5 ± 0.4	28.3 ± 0.3

Cholesterol (mmol/L)			

Total	5.75 ± 0.16	5.79 ± 0.16	5.55 ± 0.25

LDL	3.65 ± 0.15	3.64 ± 0.80	3.68 ± 0.40

HDL	1.54 ± 0.05	1.62 ± 0.06	1.20 ± 0.01

Triacylglycerols (mmol/L)	1.33 ± 0.11	1.17 ± 0.05	2.09 ± 0.20

Apolipoproteins (g/L)			

Apo A-1	1.64 ± 0.03	1.68 ± 0.04	1.42 ± 0.07

Apo B	0.96 ± 0.04	0.96 ± 0.02	0.96 ± 0.11

Insulin (mU/L)	6.75 ± 0.66	6.06 ± 0.21	9.87 ± 0.01

Glucose (mmol/L)	5.20 ± 0.06	5.18 ± 0.11	5.32 ± 0.03

HOMA-IR	1.41 ± 0.15	1.25 ± 0.07	2.16 ± 0.01

HbA_1c _(%)	4.58 ± 0.05	4.59 ± 0.03	4.53 ± 0.05

Free fatty acids (mmol/L)	0.39 ± 0.02	0.41 ± 0.07	0.35 ± 0.05

CRP (mg/L)	2.58 ± 0.34	2.79 ± 0.22	1.61 ± 0.15

IL-6 (ng/L)	0.82 ± 0.07	0.82 ± 0.03	0.81 ± 0.13

TNF-α (ng/L)	10.51 ± 0.36	10.55 ± 0.35	10.38 ± 2.00

PAI-1 (U/mL)	24.56 ± 2.28	22.83 ± 0.31	30.63 ± 2.67

Blood Pressure (mm Hg)			

Systolic	138.5 ± 2.8	139.1 ± 4.0	136.3 ± 1.2

Diastolic	80.3 ± 1.3	80.5 ± 2.0	79.8 ± 7.5

^a^Values are means ± SEM

^b ^Thirty-five post-menopausal, 1 peri-menopausal.

### Study Protocol

The study was designed as a randomized, controlled, crossover trial of the effect of a multifunctional AD on markers related to cardiometabolic risk. Participants were randomly assigned to one of two treatment orders starting with the AD or the CD, respectively. Each diet phase lasted 4 wk with a 4-wk washout period. The whole trial included four clinical visits, one before and one after each intervention period. During the first visit the participants underwent a physical examination, including auscultation of heart and lungs.

On each clinical visit, fasting body weight was recorded and BP was measured twice, while seating, in the non-dominant arm with a mercury sphyngomanometer. Venous blood was then drawn for the assessment of fasting blood glucose, insulin, HbA1c, cholesterol (total, LDL and HDL), triglycerides, high sensitivity CRP (hs-CRP), PAI-1, IL-6, TNF-α, FFA, apo A1, apo B.

Consumption of dietary supplements such as fish oil (9 subjects), probiotic-containing products (11 subjects) and herbal extracts (11 subjects), was stopped 2 weeks before the start of the trial. Before each dietary phase, the participants attended an introductory session with the nutritionist, who explained practical details of the upcoming diet period.

Volunteers were asked to maintain their normal physical activity regime during the study. A pretrial questionnaire indicated that 48% of the subjects had physical activity equivalent to 1 h/day or more, 30% had 30-60 min/day and 22% reported a low activity level (< 30 min/day). Subjects were also instructed to record their fasting body weight weekly. Variations larger than 1 Kg were reported to the nutritionist, who suggested compensatory dietary modifications. The intake of key components in AD was not affected by these modifications.

Except for almonds and fresh, frozen and smoked fish, the active food items required for the 4-wk AD were provided. Some of the foods included in the CD were also provided. In order to assess dietary compliance, participants completed a daily menu checklist covering each 4-wk diet period. They also completed a questionnaire investigating their experience with each diet. Coaching support was provided by the nutritionist, who contacted each participant at least once per diet period. The study was approved by the Regional Ethical Review Board, Lund, Sweden (Dnr 593/2008).

### Diets

Participants ate their habitual diet before and between the experimental periods. A dietary habit questionnaire completed before randomization to the starting dietary treatment was used to help the participants to resume their eating habits during the washout period. To ensure good compliance the participants were provided with a detailed 2-wk rotating menu plan for each dietary period (AD and CD) with all foods ingredients expressed in weight and/or volume measures. Electronic self-taring scales were made available whenever needed. The menu plan also included recipes for preparing meals.

The nutritional profiles of CD and AD are shown in Table 2. Both diets were designed in close agreement with the Nordic Nutrition Recommendations [22] and supplied 2,500-2,600 Kcal/d for men and 2,000-2,100 Kcal/d for women, combining foods from plant and animal origin. Both diets incorporated commercial foods available in food stores, but the AD also included prototypes. For a detailed list of products included in AD see additional file 1.

**Table 2:** Nutritional profile of CD and AD

	**Control Diet**		**Active Diet**	
	
	**Women**	**Men**	**Women**	**Men**
Energy (kcal/day)*	2045	2570	2100	2615
Protein (E%)	15	14	19	18
Carbohydrate (E%)	56	55	51	50
Fat (E%)	29	30	31	31
Saturated fat (E%)	12.8	13.2	5.9	5.9
Monounsaturated fat (E%)	10.5	11.1	13.0	13.6
Polyunsaturated fat (E%)	3.6	3.7	8.2	8.4
ω-6 fatty acids (E%)	2.9	3.1	4.2	4.3
ω-3 fatty acids (E%)	0.8	0.8	2.2	2.3
ω-6/ω-3 ratio	3.8	3.8	1.9	1.9
Dietary fiber (g/day)	22	26	49	61
Cholesterol (mg/day)	200	240	140	160

* Increased energy intake was prescribed for subjects that experienced weight loss during the initial weeks (see Study Protocol section)

AD combined several functional concepts with potential to modulate different physiological variables related to the inflammatory tonus and cardiometabolic risk, including:

a) food items naturally rich in antioxidants which, in addition to the anti-inflammatory action of their antioxidants [16], contain phenolics that may improve BP and blood lipids [23-25].

b) omega-3 fatty acids, especially those long-chained present in oily fish, which have antiinflammatory and triglyceride-lowering properties [17,26]. In addition, the overall fat quality was also a concept included in the diet design. Thus, the unsaturated-to-saturated fat ratio was larger in AD than in CD (3.6 vs 1.2, respectively; Table 2).

c) ingredients with potential to beneficially affect the gut microbiota: a probiotic strain (*Lactobacillus plantarum *Heal19, DSM 15313) [18], and prebiotics, i.e. beta-glucans and resistant starch [19,20] in intact barley kernels, whole kernel rye flour and isolated barley fiber, which were used for baking an experimental beta-glucan-rich bread. Additional sources of viscous fermentable dietary fibre were a prototype oat-based fiber drink, a rye/oat breakfast cereal and an oat-based muesli.

d) low glycemic impact foods/meals were included for their association with reduced risk for the MetS [27] and Type-2 diabetes [28], and its perceived ability to ameliorate the inflammatory tonus in healthy individuals [29]. Low GI foods were represented by products with high beta-glucan content which, besides their prebiotic role, may improve glycemic regulation in a 10-h post-ingestion perspective, through mechanisms related to colonic fermentation and lowered inflammatory tonus [20,21]. AD also included another high-fibre bread, baked from a wheat flour/guar gum blend: This item promotes a low glycemic response, with reduced peak and prolonged duration of net increment above fasting glucose levels (A. Nilsson, K. Radeborg et al., unpublished results). Additionally, AD included ingredients that lower the post-meal glycemic excursion, such as whey protein and vinegar [14,15].

e) ingredients with acknowledged abilities to normalize blood levels of both total and LDL cholesterol were also provided: different soybean products, a margarine enriched in stanol esters and dry almonds [4,12,30-32].

The mean daily quantities of the different functional ingredients in AD and the main functional properties considered for their selection are summarized in Table 3. None of the active ingredients were included in the CD, except for minor amounts of ω-3 fatty acids.

**Table 3:** Proposed functional action, previously tested dose and actual average content of active components in the active diet

	Main functional role	Previously tested dose (g/day)	References	Content in the AD (g/day)	
				
				Women	Men
Soybean/soy protein	Cholesterol-lowering, anti-inflammatory	25-47	[4,30,33-35]	21	25
Viscous fibers	Cholesterol- lowering, prebiotic, GI-reducing	10-25	[12,19]		
β-glucans				5.8	6.2
Guar gum				5.6	6.7
Total				11.4	12.9
Long chain ω-3 fatty acids (20:5 + 22:6)	Triglyceride-lowering, anti-inflammatory	2-6	[17,26]	2.4	3.0
Almonds	Cholesterol-lowering	28-100	[4,32]	28	28
Plant stanols	Cholesterol-lowering	2-3	[4,31]	2.0	2.7
Cinnamon	Antioxidant	6	[25]	3.0	3.0
Blueberries	Antioxidant, prebiotic	350	[36]	74.5	94.5
Vinegar	GI -reducing	20	[14]	22.5	22.5
Probiotic	Cholesterol-lowering, anti-inflammatory	10^10 ^CFU	[18,37,38]	0.1 (10^10 ^CFU)	0.1 (10^10 ^CFU)
Whey protein*	GI-reducing	10	[15]	4.3	4.3

*Whey protein (10 g) was only consumed simultaneously with high glycemic index meals (potatoes, parsnip), 3 times per week.

Participants were provided with a 14-day rotating menu plan. For representative 1-day menu plans for the CD and AD see additional file 2. A limited amount of alcohol-containing drinks were allowed during both dietary periods (30 and 37 g ethanol/wk for women and men, respectively). These limits, however, did not compel low-drinkers to increase their habitual alcohol consumption. Due to the low overall energy contribution from such contingent alcoholic drinks, they were not included in the estimation of the energy content of diets. The volunteers' coffee and tea drinking habits were not modified during the trial.

### Analyses

Routine blood tests were analyzed at the Clinical Chemistry Laboratory/Skåne University Hospital Lund, on fasting plasma (total and HDL cholesterol, triacylglycerols, apo A-1, apo B, high-sensitivity C-reactive protein), serum (insulin) or on total blood samples (HbA1c). LDL cholesterol concentrations were calculated [39]. HOMA-IR was calculated from fasting blood plasma-glucose and serum insulin values [40].

Venous plasma glucose concentrations were measured immediately after bleeding (HemoCue^®^B-glucose, HemoCueAB, Ängelholm, Sweden). Serum concentrations of TNF-α and IL-6 were measured with a chemiluminescent immunometric assay, Immulite^®^/Immulite^® ^1000 TNF-α (Siemens, Deerfield, IL, USA) and a sensitive enzyme-linked immunosorbent assay, Quantikine^® ^HS (RD Systems Inc, Abingdon, UK), respectively. Samples with high IL-6 concentration (> 10 ng/L) in the ELISA were also measured with the Immulite^®^/Immulite^®^1000 IL-6 assay (Siemens, Deerfield, IL, USA). Serum FFA were assessed with an enzymatic colorimetric assay kit (Wako Chemicals GmbH, Germany). Plasminogen activator inhibitor (PAI-1) activity in plasma was measured with TriniLize PAI-1 Activity kit (Trinity Biotech, Jamestown NJ, USA).

The diet compositions were analyzed using the 2009 food database from the Swedish National Food Administration and a computerized calculation program (Dietist XP 3.1; Kost och Näringsdata AB, Bromma, Sweden).

### Calculations and statistical analysis

The results are expressed as means ± SEM. Data were evaluated by mixed model ANOVA with sequence and interaction between diet and start/end of treatment periods as fixed effects, and participants within sequence and visit as random effects. Least square means were estimated for the start (Active-week 0, Control-week 0) and end values (Active-wk 4, Control-wk 4) for each diet. Least square means and confidence intervals were calculated for the differences between Active/wk 0, Control/wk 0, Active/wk 4 and Control/wk 4, as well as for the net difference between the diets, i.e. (Active/wk 4 - Active/wk 0) - (Control/wk 4 - Control/wk 0). Another set of analyses with body weight included as a continuous covariate was performed. Analyses were carried out using SAS PROC Mixed (v. 8.2, SAS Institute Inc., Cary, NC, USA). Ten-year coronary heart disease risk was calculated both with the Framingham Study equation [41] considering age, gender, total cholesterol, HDL-cholesterol, smoking and systolic BP values, and the Reynolds Risk Score [42,43], which also incorporates CRP values.

### Power calculation

Primary outcome measure was change in plasma LDL cholesterol. Assuming a 0.5 mmol/L (approximately 10%) post-diet difference and a 0.97 SD [44], with α = 0.05 and 1-β = 0.8, a minimum of 30 participants were required. Among additional measures, changes in hs-CRP were also taken into account. Assuming a 0.44 mg/L (10%) post-diet difference and a SD of 1.0 [45], the required sample size was 42 subjects. Data obtained from hypercholesterolemics with normal CRP levels [7] confirmed that this *n *value provides sufficient power for assessing CRP changes.

## Results

### Study population and dietary compliance

Recruitment began February 2009. Trials took place between March 2009 and February 2010. Forty-six volunteers enrolled in the study. The volunteers came from different towns and rural areas in Southwestern Sweden. One participant dropped out during the first week. Forty-five completed the two phases of the intervention. One completer declared to have suffered a minor cerebro-vascular incident some years ago and was therefore excluded from the evaluation. Results from 44 completers (36 women and 8 men) aged 51 - 73 (mean 63.3) were analysed. Only 2 subjects were smokers.

Baseline data confirmed the healthy condition of the cohort studied. This was also evident from the fact that 26 out of the 44 completers (i.e. 59%) showed none of the MetS features assessed (total cholesterol > 150 mg/dL, BP > 130/85 mm Hg and HDL-cholesterol < 50 mg/dL) [46].

Adherence to the dietary plan was good, with similar compliance (*P *= 0.07) for AD (92 ± 2%) and CD (89 ± 5%). No major problem to consume the prescribed foods was reported. Fifty percent of the participants described AD as more satiating than their habitual diet and 25% expressed that sensation for CD. Ninety percent of the participants experienced increased intestinal gas production with AD as compared to their regular regimes; however, only five participants (11%) qualified this as a burden.

### Body weight

Despite the dietary advice provided, weight losses were registered after CD (-0.9%) and AD (-1.8%) (Table 4). Although final body weight was similar with the two treatments, there was a significant difference between diets (*P *= 0.0113) (Table 4).

**Table 4:** Effect of CD and AD on plasma lipids, parameters of insulin sensitivity and blood pressure^a^

	**Control Diet**			**Active Diet**				
	
	**Week 0**	**Week 4**	**Change (%)**	**Week 0**	**Week 4**	**Change (%)**	***P *(A-C)**^**b**^	***P *(A-C; weight corrected)**^**c**^
	
Body weight (kg)^d^	78.9 ± 1.7	78.2 ± 1.34^h^	-0.9	79.4 ± 1.36^i^	78.0 ± 1.30^f^	-1.8	0.0113	
Cholesterol (mmol/L)								
Total^d^	5.73 ± 0.16	5.79 ± 1.39	1	5.81 ± 0.17	4.29 ± 0.10^f^	-26	< 0.0001	< 0.0001
LDL^d^	3.66 ± 0.15	3.70 ± 0.16	1	3.65 ± 0.16	2.42 ± 0.10^f^	-34	< 0.0001	< 0.0001
HDL^e^	1.53 ± 0.04	1.43 ± 0.04^h^	-7	1.54 ± 0.05	1.39 ± 0.04^g^	-10	0.1637	0.1501
Triglycerides (mmol/L)^d^	1.36 ± 0.11	1.38 ± 0.62	1	1.32 ± 0.09	1.07 ± 0.06^g^	-19	0.0056	0.0011
Apolipoproteins (g/L)								
Apo A-1^d^	1.62 ± 0.03	1.54 ± 0.03^f^	5	1.62 ± 0.04	1.40 ± 0.02^f^	-14	< 0.0001	< 0.0001
Apo B^d^	0.95 ± 0.04	0.95 ± 0.03	0	0.94 ± 0.04	0.72 ± 0.02^f^	-23	< 0.0001	< 0.0001
Ratios								
LDL/HDL^d^	2.53 ± 0.03	2.73 ± 0.03^h^	8	2.51 ± 0.02	1.83 ± 0.03^f^	-27	< 0.0001	< 0.0001
ApoB/ApoA-1^d^	0.60 ± 0.03	0.63 ± 0.03	5	0.59 ± 0.03	0.53 ± 0.02^f^	-10	< 0.0001	< 0.0001
Insulin (mU/L)^e^	6.70 ± 0.42	5.70 ± 0.25	-14	6.08 ± 0.42	5.03 ± 0.36^h^	-17	0.9410	0.9826
Glucose (mmol/L)^d^	5.21 ± 0.07	5.13 ± 0.07	-1	5.17 ± 0.07	5.46 ± 0.08^g^	6	0.0002	0.0002
HOMA-IR	1.39 ± 0.08	1.17 ± 0.08	-16	1.26 ± 0.09	1.09 ± 0.06	-14	0.7376	0.6983
HbA_1c _(%)^d^	4.56 ± 0.05	4.58 ± 0.05	1	4.55 ± 0.05	4.47 ± 0.05^h^	-2	0.0007	0.0013
Free fatty acids (mmol/L)	0.37 ± 0.02	0.37 ± 0.02	0	0.37 ± 0.02	0.37 ± 0.03	0	0.9673	0.9627
Blood pressure (mm Hg)								
Systolic^e^	135 ± 2	132 ± 2	-2	136 ± 2	128 ± 2^f^	-8	0.0123	0.0134
Diastolic	79 ± 1	76 ± 1	-4	79 ± 1	76 ± 1	-4	0.9984	0.9265

^a ^Values are means ± SEM. To convert cholesterol and triglyceride values to mg/dL, multiply by 38.67 and 88.57, respectively. To convert apo A-1 and apo B values to mg/dL, multiply by 100.

^b^*P *value for the comparison of changes after Active diet and Control diet.

^c^*P *value for the comparison of changes after Active diet and Control diet, after correction for weight change

^d, e ^The overall effect of diet was significant: ^d^*P *< 0.001, ^e^*P *< 0.05.

^f, g, h ^Significantly different from baseline (Week 0): ^f^*P *< 0.0001, ^g^*P *< 0.001, ^h^*P *< 0.05.

^i^Active diet baseline (Week 0) significantly different from control diet baseline, *P *< 0.05.

### Blood lipids and lipoproteins

AD reduced total cholesterol (*P *< 0.0001), LDL cholesterol (*P *< 0.0001) and triglycerides (*P *= 0.0004) by 26%, 24% and 19%, respectively, while no change was recorded after the CD period (Table 4). The difference between the two dietary treatments remained after correction for body weight reduction (*P *< 0.0001, *P *< 0.0001 and *P *= 0.0011, respectively; Table 4).

HDL-cholesterol values were similarly reduced by both diets (*P *= 0.01635) but AD improved both the LDL/HDL ratio (from 2.51 to 1.83, ie. -27%, *P *< 0.0001) and ApoB to ApoA-1 ratio (from 0.59 to 0.53; -10%; *P *< 0.0001). The difference between diets was significant (*P *< 0.0001) after correction for weight changes (Table 4).

### Glycemic control variables

No difference in the effect on fasting insulin concentrations was recorded between AD and CD (*P *= 0.9410), although AD promoted a significant (*P *= 0.0436) reduction from baseline (Table 4). AD increased fasting blood glucose levels by 6%, with a significant difference between diets after correction for weight changes (*P *= 0.0002; Table 4). None of the diets affected HOMA-IR values or fasting FFA levels. A 2% decrease in HbA1c was detected after AD, an effect that remained significant after correction for body weight variation (*P *= 0.0013; Table 4).

### Blood pressure

Systolic BP was 8% lower after AD whereas CD had no effect. The difference between diets remained significant after weight correction (*P *= 0.0134; Table 4). Diastolic pressure values were not affected by any of the diets (Table 4).

### Inflammatory and pro-thrombotic status markers

AD reduced hs-CRP value by 29%, with no effect for CD (Table 5). The difference between diets was significant after correction for body weight reduction (*P *= 0.0497). In spite of a 26% drop in PAI-1 after AD, no net difference was registered between the two diets (*P *= 0.3854). None of the diets affected fasting IL-6 or TNF-α concentrations.

**Table 5:** Effect of CD and AD on circulating inflammatory and thrombotic markers^a^

	**Control Diet**			**Active Diet**				
	
	**Week 0**	**Week 4**	**Change (%)**	**Week 0**	**Week 4**	**Change (%)**	***P *(A-C)**^**b**^	***P *(A-C; weight corrected)**^**c**^
	
CRP (mg/L)	2.59 ± 0.34	2.89 ± 0.42	12	2.79 ± 0.39	1.99 ± 0.27^e^	-29	0.0446	0.0497
IL-6 (ng/L)	0.96 ± 0.09	0.93 ± 0.09	-3	0.71 ± 0.05^d^	0.81 ± 0.09	14	0.6232	0.3807
TNF-α (ng/L)	9.95 ± 0.36	10.09 ± 0.45	1	11.04 ± 0.46^d^	10.38 ± 0.42	-6	0.0971	0.1094
PAI-1 (U/mL)	23.34 ± 1.69	19.33 ± 1.68	-19	23.70 ± 2.17	17.65 ± 1.47^e^	-26	0.3854	0.4104

^a^Values are expressed as means ± SEM.

^b^*P *value for the comparison of changes after Active diet and Control diet.

^c^*P *value for the comparison of changes after Active diet and Control diet, after correction for weight change

^d^Active diet baseline (Week 0) significantly different from control diet baseline, *P *< 0.05.

^e^Significantly different from baseline (Week 0), *P *< 0.05.

### Cardiovascular risk

Significantly lower overall cardiovascular risk scores were calculated after AD (Table 6). The Framingham Study algorithm indicated a 30-percent risk drop (*P *< 0.0001). The Reynolds risk score predicted a larger risk decline (35%; *P *< 0.0001). The CD did not reduce risk estimates. The reduction in risk estimates with AD remained significant after weight correction.

**Table 6:** Effect of CD and AD on 10-y cardiovascular risk^a^

	Control Diet			Active Diet			***P *(A-C)**^**b**^	***P *(A-C; weight corrected)**^**c**^
	
Calculated 10-y CHD risk (%)	Week 0	Week 4	Change (%)	Week 0	Week 4	Change (%)		
Framingham equation^d, f^	5.0 ± 0.8	5.2 ± 0.8	4	5.2 ± 0.8	3.6 ± 0.6^e^	-30	< 0.0001	0.0002
Reynolds score^d, g^	6.2 ± 0.8	6.2 ± 0.8	1	6.3 ± 0.9	4.1 ± 0.6^e^	-35	< 0.0001	< 0.0001

^a ^Values are means ± SEM.

^b^*P *value for the comparison of changes after Active diet and Control diet.

^c^*P *value for the comparison of changes after Active diet and Control diet, after correction for weight change.

^d ^The overall effect of diet was significant: *P *< 0.0001.

^e ^Significantly different from baseline (Week 0), *P *< 0.0001.

^f ^CHD risk calculated using the Framingham cardiovascular disease predictive equation [41] considering gender, age, total cholesterol, HDL-cholesterol, and smoking habits.

^g ^CHD risk calculated using the Reynolds risk score [42,43] considering gender, age, total cholesterol, HDL-cholesterol, CRP and smoking habits.

## Discussion

This investigation looked at the possibility of modulating diverse cardiometabolic risk factors by a diet based on multiple functional food concepts. Although a number of therapy-oriented dietary studies have been reported in dyslipidemic subjects [4,6,8,45,47], no randomized intervention has examined the potential CMD preventive power of a complex functional food array targeting subclinical inflammation. Present results put forward interesting possibilities for this dietary approach.

One characteristic of this study is the healthy condition of the cohort evaluated. The volunteers had no particular diagnosis, beyond being considered at risk for cardiometabolic alterations due to their age (63.3 ± 0.8 y) and BMI (28.5 ± 0.3 kg/m^2^). According to mean baseline values for blood lipids, BP and glucose homeostasis parameters (Table 1), the group represents the healthy segment of the Swedish population in the 50-75 year-old range. This judgment is supported by the fact that 59% of the volunteers exhibited none of the MetS features evaluated, which contrasts with data from a recent survey of MetS prevalence among 50-60 year olders living in Gothenburg, showing that only 5% of this population group has no risk factor associated to the syndrome [48].

The AD evaluated here included a cluster of functional concepts selected for their abilities to influence different CMD-associated factors, with emphasis on anti-inflammatory properties. The efficacy of the individual concepts has been documented at various levels including intervention studies with humans. Furthermore, since the concepts influence different aspects of the pathophysiological processes associated with CMD, synergistic interaction between functional agents may occur and facilitate the overall modulation of multiple risk indicators. Thus, the daily dosage of some functional components was set below the amount required for optimal effect when tested separately (Table 3), improving the general palatability of the diet.

CD exhibited a good nutritional profile, which matched the Nordic Nutritional Recommendations. Although its dietary fiber content was lower, the CD contribution approached the current estimated fiber intake for the Swedish population (Swedish Food Administration, Personal Communication). Also, besides being rich in functional ingredients, AD had an improved fat quality (Table 2) and its long-chain ω-3 fatty acid content was 10 times larger than in CD. Both diets were well accepted and tolerated by the participants. The variety of foods encompassed, the inclusion of meat and other animal products and the minor amount of alcoholic drinks allowed favored the high dietary adherence and completion rates recorded.

A somewhat greater weight loss was observed after AD, which agrees with the marked satiating action reported for this diet by most participants, an effect that may be due to its higher dietary fiber and protein contents. In fact, 50% of the participants were prescribed increased dietary energy after the first 2 weeks on the regime when their weight loss tendency became evident, in contrast to only 25% requiring that intervention during the CD period. In spite of that a 1.8% (1.4 ± 0.2 kg) mean decrease was recorded after AD. Nonetheless, the statistical adjustment made for the impact of weight variation showed that most of the metabolic improvement observed after AD was attributable to the dietary modification *per se *and not to a mere weight loss effect.

Blood lipid and apolipoprotein profiles were practically unaffected by CD but sharp reductions in triglycerides, total cholesterol, LDL cholesterol as well as in LDL/HDL and apoB/apoA1 ratios were recorded after the AD period. The latter observation is noteworthy, given the strong association between this quotient and the MetS [49]. The beneficial impact of a low glycemic index diet on blood cholesterol [50] together with the triglyceride-reducing action of polyunsaturated fatty acids from marine origin [10] and content of other known cholesterol-lowering foods and ingredients (soybeans and soy protein, viscous dietary fibers, plant stanols and almonds) are most probably responsible for the overall effect observed with AD.

None of the diets affected insulin sensitivity as estimated by HOMA-IR, although AD promoted slightly higher fasting glucose values than CD. Such an increased glycemia should not pose a risk for healthy individuals, a presumption supported by the reduction recorded in HbA_1c _and unchanged values for fasting FFA.

The minor decrease in HbA_1-c _observed with AD might be the consequence of modulated glycation rate during the AD period. The diet's low glycemic impact and high phenolic content, both with anti-glycative effects [51,52], may have contributed to this result.

CRP is an indicator of the inflammatory status of recognized importance in relation to cardiometabolic alterations [42,53], with independent predictive value for incident diabetes and cardiovascular diseases [7,54]. The biomarker decreased markedly after AD, with significant difference between diets after adjustment for weight change. It is tempting to speculate that the improved CRP levels may be due to the antinflammatory properties of AD. The CRP-lowering effectivity of a diet enriched in oily fish, blueberries and wholegrain products was recently shown in patients with impaired glucose tolerance [55].

In addition to improving metabolic risk indicators, AD promoted a significant drop in systolic BP. Such a change may be related, for instance, to the high supply of long chain ω-3 fatty acids, ingredients with suggested BP-modulating activity [56]. Although the present cohort was normotensive (Table 1), the observed pressure reduction resembles that observed with a composite drug treatment in hypertensive volunteers [57].

Besides modulating individual markers associated to CMD, AD also decreased cardiovascular risk as estimated by two different models. The Reynolds algorithm resulted in larger variation from baseline (-34%) than the Framingham score (-30%), stressing the importance of the CRP reduction promoted by this diet.

The improvement achieved for different biomarkers after AD in this relatively low risk group, compares favorably with other dietary interventions. Most of the investigations on the effect of diet CMD-related parameters have been carried out in patients that have metabolic alterations, such as dyslipidemia. A portfolio of cholesterol-lowering foods reduced LDL cholesterol values by 33% in a 4-week trial with hypercholesterolemics [5], which is alike the variation recorded here with a normocholesterolemic group. Furthermore, the presently reported impact of AD on the apoB/Apo A1 ratio, CRP concentration and Framinghams CVD risk is comparable to those previously attained in hypercholesterolemics following the cholesterol-lowering portfolio regime [5,6].

Also, the effect of AD on total (-26%) and LDL cholesterol levels (-34%) exceeds those reported for the "Nordic" diet in hypercholesterolemic individuals (-16% and -21%, respectively) a regime that, in contrast to ours, did not affect triglycerides or systolic BP beyond weight-loss effects [8]. The AD also promoted greater LDL-cholesterol, triglyceride and CRP reductions than Mediterranean-style regimes administered to individuals at high cardiovascular risk [58].

Compared to other dietary interventions performed in healthy volunteers, the action of the AD on different CMD-related variables is also remarkable. Its effects on systolic BP are similar to those of reduced sodium/DASH [59] or whole-grain [60] regimes. Moreover, in terms of LDL cholesterol reduction AD appears impressively more effective than vegetarian [61] or low GI [50] regimes. The larger effectiveness of AD reflects the power of its multifunctional character.

It is relevant noting that the AD effects were observed under conditions aiming to prevent weight changes. In view of the perceived high satiating ability of the diet, larger weight reductions could be expected if it was consumed ad libitum. This potential deserves further investigation, since dietary interventions promoting weight losses of 5% or more generally result in additional metabolic improvement [9,62,63].

The present study had limitations. An evident constraint is the unbalanced gender participation, since 80% of the participants were women. Nevertheless, the lower responsiveness of women to treatments targeting metabolic alterations, such as hyperlipidemias [64,65], stresses the relevance of our results. The relative heterogeneity of the cohort regarding metabolic characteristics may also be seen as limiting. However, the group reflects the healthy mature segment of the local population. The study length represents another limitation. Prolonged open interventions often yield inferior results, a pattern influenced by reduced dietary compliance. Nonetheless, the good acceptability recorded for the AD allows expectations for reasonable treatment adherence in longer term trials. An increased availability of active food items and ingredients in the near future may ease the achievement of this goal.

The possible impact of AD on body composition and fat distribution was not evaluated here. Since these factors are importantly associated with cardiometabolic risk [46,54], it would be interesting to include them in future studies on the role of functional diets in the prevention of cardiometabolic alterations. At last, the experimental design envisaged sufficient statistical power regarding changes in LDL cholesterol and CRP but the greater statistical dispersion associated to other biomarkers, such as insulin, PAI-1 and TNF-α, may have left the study underpowered to confirm their modification. This should be born in mind when interpreting the non-significant changes recorded in these parameters.

## Conclusion

Tested in a group of middle-aged healthy overweight individuals, a multifunctional diet could modulate different CMD-related variables. The beneficial metabolic effects, good acceptability and ease of implementation shown for the diet in this cohort, suggest this type of regime as a promising tool for dietary preventive action against CMD.

## Abbreviations

AD: active diet; apoA: apolipoprotein A; Apo B1: apolipoprotein B1; BP: blood pressure; CD: control diet; CMD: cardiometabolic diseases; CRP: C-reactive protein; FFA: free fatty acids; HBA1c: glycated hemoglobin; HDL: high density lipoprotein; hs-CRP: high sensitivity C-reactive protein; HOMA-IR: homeostatic model assessment of insulin resistance; IL-6: interleukin 6; LDL: low density lipoprotein; MetS: Metabolic syndrome; PAI-1: plasminogen activator inhibitor 1; TNF-α: tumor necrosis factor alpha.

## Competing interests

The authors declare that they have no competing interests.

## Authors' contributions

IB, JT and MJ were responsible for the study concept and design; JT, A-MÅ and AN conducted the research; UJ designed the menus; UJ and JT were responsible for the participant's dietetic instruction and coaching; RE was the medical supervisor of the study; JT. and AN analyzed data; JT drafted the manuscript; JT and IB had primary responsibility for final content. All authors revised, discussed and approved the final paper.

## Supplementary Material

Additional file 1 **Functional products and ingredients included in the active diet**. Click here for file

Additional file 2 **Representative 1-day menus for AD and CD**. Click here for file
