# Supplementary material for: A diet based on multiple functional concepts improves cardiometabolic risk parameters in healthy subjects
Source: Nutr Metab (Lond). 2012 Apr 2;9:29. doi: 10.1186/1743-7075-9-29 (PMC3361470; doi:10.1186/1743-7075-9-29)
Supplement: Additional file 1 — Functional products and ingredients included in the active diet. [file 1743-7075-9-29-S1.PDF]

ADDITIONAL FILE 1

Functional products and ingredients included in the active diet:

a) Antioxidant-rich items: frozen blueberries (*Vaccinium myrtillus*) were purchased from ICA Sweden AB (Solna), blueberry purée was a product from Semper AB (Sundbyberg, Sweden) and ground cassia cinnamon was provided by Lyckeby Culinar AB (Fjälkinge, Sweden). The cinnamon preparation contained coumarin levels under 2000 ppm.

b) Sources of omega-3 fatty acids: canned brisling and mackerel (Abba Seafood AB, Gothenburg, Sweden) were sources of long chain fatty acids and cold-pressed rapeseed oil (Dr PersFood AB, Eslöv, Sweden) provided the shorter chained counterpart.

c) Probiotic: *Lactobacillus plantarum* Heal19 (DSM 15313), was provided in a dosed powdered form (10^{10} CFU/day; Probi Ltd, Lund, Sweden). The strain ability to metabolize tannins (G. Molin, personal communication) was seen as an advantage in a phenolic-rich regime, such as AD.

d) Prebiotics and glycemic response-modulating ingredients: Intact barley kernels, whole rye flour and isolated barley fiber used for baking the beta-glucan rich bread were provided by Lyckeby Culinar AB, Fjälkinge, Sweden. This bread provided a total of 2.3 g beta-glucans/100 g, fresh basis. The guar gum-containing bread provided 6.4 g/100 g guar gum (fresh basis). These two prototype breads were baked by Credin A/S (Juelsminde, Denmark). Additional sources of viscous soluble dietary fibre were a prototype oat-based fiber drink providing 0.8 g beta-glucan per 100 g (Oatly AB, Landskrona, Sweden), a rye/oat breakfast cereal (Quaker Oats Sweden, Malmö,

Sweden) and an oat-based muesli (12 g beta-glucan/100g; Prorsum Healthcare AB, Gothenburg, Sweden). Powdered whey protein was from Lyckeby Culinar AB (Fjälkinge, Sweden) and Dr PersFood AB (Eslöv, Sweden) provided a salad dressing (supplying 6 g vinegar/100 g).

e) Cholesterol and triglyceride lowering ingredients: soy protein in the form of soy-based balls, burgers and mince (Hälsans Kök-Tivall (Helsingborg, Sweden), tofu (Kung Markatta, AB (Hjälmarsberg, Sweden), soy-based yogurt (Raiso Sverige AB, Solna, Sweden) and canned whole soybeans (Zeta-Di Lucca AB, Stockholm, Sweden), and margarine enriched in stanol esters (Benecol, Raisio Sverige AB; Solna, Sweden).
