# Supplementary material for: A diet based on multiple functional concepts improves cardiometabolic risk parameters in healthy subjects
Source: Nutr Metab (Lond). 2012 Apr 2;9:29. doi: 10.1186/1743-7075-9-29 (PMC3361470; doi:10.1186/1743-7075-9-29)
Supplement: Additional file 2 — Representative 1-day menus for AD and CD. [file 1743-7075-9-29-S2.PDF]

ADDITIONAL FILE 2

Representative 1-day menus for AD and CD

	Active Diet	Control Diet
Breakfast		
	Soy-based yoghurt	Yoghurt/sour milk (1.5 % fat, unsweetened)
	Oat-fiber muesly	Corn flakes
	Rye/oat muesly	Apple purée
	Blueberries	
	Cinnamon	
	Probiotic culture (powdered)	
Snack		
	Guar gum bread	White wheat bread
	Stanol-containing margarine	Margarine
		Cheese (28% fat)
		Cucumber
Lunch		
	Rye-barley bread	White wheat bread
	Guar gum bread	Wheat crisp bread
	Rye crisp bread	Margarine (butter-oil blend)
	Stanol-containing margarine	Cheese (28% fat)
	Canned mackerel in tomato sauce	Smoked sausage (30% fat)
	Lean ham (max. 3% fat)	Mixed greens and vegetables
	Mixed greens and vegetables	Orange marmalade
	Cheese (17% fat)	
Dinner		
	Lean beef	Blended (pork/beef) mince
	Parsnip	Onion
	Potatoes	Margarine (butter-oil blend)
	Whey protein	Carrot
	Canned soybeans	Canned tomatoes
	Rapeseed oil	Sweet corn (frozen)
	Carrot	Pasta
	Lettuce	Parmesan cheese
	Salad dressing	Lettuce
Snack		
	Almonds (with peel)	Cookies or sweet wheatbun
	Oat-based drink	Crisp rolls (wheat flour)
	Blueberry purée	Margarine (butter-oil blend)
	Mixed fruits (min. 3/day)	Mixed fruits (min. 3/day).
